# Dynamic Linkages among Economic Development, Energy Consumption, Environment and Health Sustainable in EU and Non-EU Countries

**DOI:** 10.3390/healthcare7040138

**Published:** 2019-11-06

**Authors:** Yongqi Feng, Xinye Yu, Yung-ho Chiu, Tzu-Han Chang

**Affiliations:** 1 School of Economics, Jilin University, No. 2699, Qianjin Street, Changchun 130012, China; fyqjldx@jlu.edu.cn (Y.F.); yuxinye1024@163.com (X.Y.); 2 Department of Economics, Soochow University, No. 56, Kueiyang St., Sec. 1, Taipei 100, Taiwan; echiu@scu.edu.tw

**Keywords:** energy efficiency, health efficiency, EU countries, meta-frontier dynamic network DEA

## Abstract

There is a close and important relationship between environmental pollution and public health, and environmental pollution has an important impact on the public health. This study employed the two-stage meta-frontier dynamic network data envelopment analysis (TMDN-DEA) model to explore the environment pollution effects from energy consumption on the mortality of children and adult, tuberculosis rate, survival rate and health expenditure efficiencies in 28 EU countries and 53 non-EU countries from 2010 to 2014. We calculated the overall efficiency scores and the technology gap ratios of each EU and non-EU countries and the efficiencies of input and output variables in the production and health stage. The average overall efficiencies each year in EU countries are higher than in the non-EU countries. But EU countries have higher energy efficiency than non-EU countries, and non-EU countries have higher health efficiency than EU countries. The health expenditure efficiencies in the EU countries are obviously lower than those in non-EU countries. The renewable energy efficiencies are obviously higher than the non-renewable energy efficiencies; PM2.5 efficiencies are obviously higher than the CO_2_ efficiencies and the children’s mortality rate efficiencies are higher than the adult’s mortality rate efficiencies for EU countries and non-EU countries. The government management in the EU and non-EU countries should be strengthened to reduce the air pollutant and carbon dioxide emissions and raise energy transformation to the clean energy in renewable energy and improve health efficiencies in medical and health care field.

## 1. Introduction

The European Union (EU) is an important intergovernmental economic union and produced 21.54% GDP of the world in 2018 [1]. The EU is actively exploring the issue of climate change and the environmental pollution. The EU has set a target of 20% renewable energy in total energy use in 2020. There are 11 EU members that have reached their 2020 targets, with Sweden’s 54.5% target being the highest share of renewable energy in total energy in the EU, with Luxembourg’s 6.4% and the Netherland’s 6.6% target are the lowest. France’s goal of achieving its 2020 renewable energy share is 23%, but it has not yet been achieved [2]. 

The other countries also attach great importance to energy efficiency and environmental issues. As early as 1997, the United Nations Framework Convention on Climate Change adopted the Kyoto Protocol at the third Conference of States Parties. In November 2017, the 23rd Conference of the Parties to the United Nations Framework Convention on Climate Change was held in Bonn, Germany. The conference formulated the implementation guidelines for the Paris Agreement. The main content includes controlling the global temperature to within 2 degrees Celsius before the industrial revolution and reducing greenhouse gas emissions in stages. 

**Figure 1:**
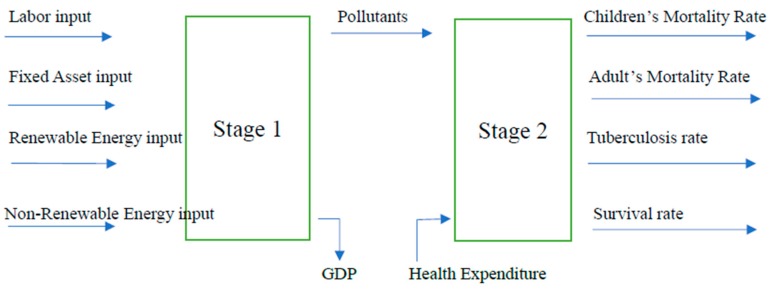
Process of inputs and outputs in production and health treatment stage.

There are some input-output relationship and influence mechanism among energy, environment pollution, and health, as shown in the Figure 1. When energy consumption and labor and capital input contribute to economic growth, they can result in environmental pollution, for example, carbon emissions and air pollution. The carbon emissions and air pollution have very strong impact on the respiratory, heart, and brain functions and lead to some serious disease, although government and society will have a lot of relational health expenditure for the health treatment.

Based on such influence and transmission mechanism, this study employed a two-stage meta-frontier dynamic network data envelopment analysis (TMDN-DEA) model to explore the environment pollution effects from energy consumption on the mortality of children and adult, tuberculosis rate, survival rate and health expenditure efficiencies in EU and non-EU countries. This research analyzes the energy and health efficiencies in EU and non-EU countries. The first stage is the production stage and we can learn the energy efficiencies from this stage. The second stage is the health treatment stage and we can learn the health efficiencies from this stage. 

This study has two main contributions. First, energy, environment, and health are included in one model to comprehensively explore energy and health efficiency of EU countries and non-EU countries taking comparative analysis. Second, this study divides the energy into the renewable energy and the non-renewable energy and divides the mortality rate into children’s mortality rate and adult’s mortality rate.

The remainder of this article is organized as follows: the second section gives the literature review, the third section introduces the research model and method, the fourth section gives the empirical study results, and the fifth section presents the conclusions and implications.

## 2. Literature Review

There have been many studies on energy environment and health issues, mainly from two independent directions of environment and health. The first area has been focusing on energy and environmental efficiency analyses. The second area is about the impact on human health from environmental pollution.

Energy and environmental topics have long been analyzed by many scholars from the perspectives of economy, energy, pollution, and governance. With regard to the importance of energy and environmental issues, many scholars have also done a lot of research on this topic. Among them, some scholars use the data envelopment analysis (DEA) method to study the energy efficiency [3,4,5,6,7,8,9,10,11,12,13,14]. The EU countries are also the important research objects of energy and environmental issues [15,16,17,18,19]. These studies focus on the relationship between energy and environment, the impact on environment from the economy, energy, pollution. One reason why energy and environmental issues are widely concerned is because it is closely related to our health. Some scholars also studied the relationship between environmental pollution and human health [20,21,22,23,24,25,26,27,28,29,30,31,32,33]. As we know, the EU countries are also the regions in the world that have invested heavily in health care. In the case of environmental pollution inevitably, what is the effect of health input in EU? This is a question worth considering and studying. However, there has been less research jointly focusing on the associations between energy, environment pollution, and health. 

Sueyoshi and Mika [3] proposed a non-oriented DEA model to study the gas (NO_X_) caused by acid rain in the United States Clean Air Act (CAA). This study found that the environmental law is effective for the emission control of SO_2_ and NO_X_ produced by coal-fired power plants in the United States. Liou and Wu [4] used the DEA model to analyze the global energy efficiency and carbon dioxide emissions and to seek pure technical efficiency to improve CO_2_ emissions control scale efficiency for utilization in developing countries. Choi et al. [5] used the SBM-DEA method to explore China’s energy efficiency and found that carbon dioxide efficiency was seriously poor. Zhang and Choi [6] used SBM-DEA to study the environmental efficiency of various provinces in China. The results showed that most provinces had low energy efficiency and there were significant differences in environmental efficiency between regions. Yang and Wang [7] used the DEA model to collect data from various provinces in China from 2000 to 2007 to explore the energy efficiency on environment. The results showed that the energy efficiency on environment in China is low and economic output and carbon dioxide emissions need to be improved. Zhao et al. [8] believed that the power industry is China’s largest source of air pollution, accounting for 40% of carbon dioxide emissions and 60% of sulfur dioxide emissions. The Chinese power plant industry must implement environmental regulations to improve the efficiency and environmental performance; moreover, reducing carbon dioxide has a significant impact. Yao et al. [9] collected panel data of China’s provincial industrial sector from 1998 to 2011, using the meta-frontier non-radial Malmquist CO_2_ emission performance index (MNMCPI) indicator to analyze the changes in China’s carbon dioxide emission efficiency and its driving force. The empirical results showed that the average annual growth rate of CO_2_ emissions from China’s provincial industrial sector was 5.53% from 1998 to 2011. The average carbon dioxide emissions of the industrial sector in the eastern, central, and western regions decreased in turn, and the annual growth rate of MNMCPI’s efficiency (EC) indicators increased. The rate was 2.297%, and the carbon dioxide emission efficiency change (EC) in 21 provinces showed an upward trend. Wang et al. [10] explored China’s energy efficiency from 2008 to 2012 by non-oriented DEA model. The results showed that Shandong and Hainan were effective in terms of natural and management disposition, while other provinces are likely to improve their energy and environmental performance. Many provincial industrial sectors should strive to reduce pollution through technology investments. In addition, the average under natural and managerial disposability in western China was highest, followed by eastern China, and central China. Qin et al. [11] used data envelopment analysis to assess the energy efficiency of China’s coastal areas from 2000 to 2012. The empirical results showed that the economic development level of China’s coastal areas was positively correlated with energy efficiency performance, except for Beijing and Hainan. Sağlam [12] explored the energy efficiency of the 39 states of the United States using a two-stage DEA model. The results showed that more than half of the states had high energy efficiency and can effectively reduce carbon dioxide emissions. Feng et al. [13] analyzed China’s total carbon dioxide emissions efficiency and carbon dioxide emission reduction potential. The results showed that because of structural inefficiency, technical and management efficiency is also low, China’s carbon dioxide emission efficiency was relatively low, and the government should rely on industry structural adjustments to reduce regional technology gaps to reduce carbon dioxide emissions. Bi et al. [14] used the SBM-DEA model to explore the relationship between fossil fuel consumption and China’s thermal power generation environmental regulation. The results showed that energy efficiency and environmental efficiency were relatively low, and the energy and environmental efficiency scores of different provinces varied widely. Mingxing Sun et al. [15] studied the pulp industry in many countries and regions, through meta-analysis, and concluded that the main factor affecting the greenhouse gas emissions in papermaking process is energy using. In pulping process, the energy utilization rate is 62%, and the greenhouse gas emissions are 45%. Shihong Zeng et al. [16] used VAR model to study the dynamic relationship among emission limitation price, economic development, and energy price in Beijing. Mojie Li et al. [17] summarized the development policy of China’s non-ferrous metal industry when they studied China’s non-ferrous metal industry. The carbon emissions of non-ferrous metal industry are analyzed by bottom-up model. Shihong Zeng et al. [18] also studied the efficiency of investment in China’s new energy industry and found that the investment efficiency of new energy enterprises is affected by both macroeconomic conditions and specific characteristics of enterprises by DEA model analysis.

There are also many studies to take European countries as research objects on energy environment and health issues. Bampatsou et al. [19] used the DEA model to explore the energy efficiency of the 15 EU countries from 1980 to 2008. The results showed that the input of nuclear energy as a mixture of energy had a negative impact on the efficiency of countries and also caused serious environmental problems. Cucchiella et al. [20] used the DEA model to explore the energy and environmental efficiency of EU countries. Research and results showed that the energy and environmental efficiency of the EU countries were low, and countries with poor efficiency can influence energy efficiency through potential emissions and energy consumption reduction. Gomez-Calvet [21] used the directional distance function to analyze the energy efficiency of 25 EU countries. The results show that there are significant efficiency differences between EU countries, especially in the latest EU countries. The energy efficiency is poor and environmental policies need to be formulated to reduce CO_2_ emissions. Dumana and Kasman [22] used the parametric hyperbolic distance function to study the environmental efficiency of EU Member States during the period 1990-2011. The results showed that the environmental technical efficiency scores among EU countries were different, compared with new members and candidate countries. The first 15 countries in EU had greater potential to reduce carbon dioxide emissions while increasing gross domestic product and reducing energy use. Cecchini et al. [23] used the DEA model to explore the energy efficiency of European livestock industry. The results showed that the improvement of European livestock technology had a significant relationship to reduce carbon dioxide emissions. Suzuki and Nijkamp [24] found EU countries appear to exhibit generally a higher energy-environment-economic efficiency than APEC and ASEAN countries by distance friction minimization (DFM) model. Moutinho et al. [25] used both data envelopment analysis and stochastic frontier analysis to compute the agriculture technical efficiency scores of 27 European countries. Reinhard et al. [26] estimated the comprehensive environmental efficiency measures for Dutch dairy farms by stochastic frontier analysis (SFA) and data envelopment analysis (DEA). Robaina-Alves and Moutinho V. [27] identified the effects the intensity of GHG emissions (El) in agriculture and analyzed which of them has more importance in determining the intensity of emissions in agriculture for European countries. Toma et al. [28] examined the agricultural efficiency of EU countries through a bootstrap-data envelopment analysis (DEA) approach and indicated that most of the oldest EU countries had a more efficient and optimized crop production process in terms of resource savings and output maximization. Vlontzos G., Niavis S., Manos B. [29] evaluated the energy and environmental efficiency of the primary sectors of the EU Member State countries based on a non-radial data envelopment analysis (DEA) model and found a series of eastern European countries achieve low efficiency scores. Some scholars have also explored the impact of exposure to environmental pollution on human health. Cohen et al. [30] found that the mortality from PM2.5 increased from 35 million in 1990 to 42 million in 2015. Wang [31] explored the impact of energy consumption emissions from various industries and regions in China on population health hazards. Studies have shown that increased PM10 and SO_2_ emissions can significantly harm population health. Fischer et al. [32] explored long-term exposure to air pollution and urban studies and concluded that long-term exposure to PM10 and NO_2_ in the Netherlands over 30 years of age is associated with increased mortality. Yang et al. [33] studied the effects of long-term exposure to ambient air pollution on hypertension and analyzed 24,845 adults (aged 18–74 years) in three cities in Northeast China in 2009. The results showed that pollutants and hypertension were positively correlated in the early stage and also had significant effects on systolic and diastolic blood pressure, and long-term exposure to environmental air pollution is associated with pre-hypertension and high blood pressure, especially in women and the elderly. Li et al. [34] explored air pollution and health problems in various provinces in China. The research showed that PM2.5 emissions and economic losses caused different health problems. It was also found that Beijing’s energy consumption and PM2.5 emissions both showed rapid growth and mortality. The economic losses were the biggest, and the government should reduce PM2.5 emissions to reduce public health impacts. Liu et al. [35] used the LEAP (long-range energy alternative planning system) model to analyze the carbon dioxide emissions and health problems caused by energy consumption from 2010 to 2015. The results showed that acute bronchitis was the most serious affected by PM10 pollution, and policy measures should be taken to reduce carbon dioxide and pollutant emissions. Dauch et al. [36] explored the relationship between short-term exposure to air pollution and lung function in northern French cities, and the relationship between non-respiratory adult non-respiratory diseases in middle-aged non-smoking adults. The results showed that O_3_ increased in the blood. There was a significant relationship between the increase in eosinophil count, and the clinical decline in healthy lung function and the increase in inflammatory markers in French residents with short-term exposure to air pollution. Carlton et al. [37] assessed the relationship between air exchange rates and respiratory health in a multi-ethnic population living in low-income urban households, using a structured questionnaire from a standard instrument to estimate the annual average air exchange rate (AAER) for each family. Correlation with respiratory symptoms had shown that residents in families with higher AAERs were more likely to suffer from chronic cough, asthma, and asthma-like symptoms. Shen et al. [38] used the total air quality index (AQI) and the health risk air quality index (HAQI) to assess health risks. The results showed that current AQI systems may significantly underestimate the health risks of air pollution based on HAQI results. The public may need stricter health protection measures to ensure safety. Ljungman et al. [39] used linear regression to study the relationship between long-term and short-term air pollution exposure and arterial stiffness. The results showed that long-term exposure to PM2.5 was not associated with arterial stiffness but was positively correlated with life near the main road, indicating that the contaminant mixture was very close to the main road, not PM2.5, which may affect the arterial stiffness. In addition, short-term air pollution exposure was not associated with higher arterial stiffness. Torres et al. [40] studied exposure and its adverse health effects. The results showed that sulfur dioxide and fine particles in the Alentejo and Lisbon metropolitan areas showed an increasing trend, with deaths in the northern regions and metropolitan areas. The rate had also increased significantly. Chen et al. [41] explored the effects of short-term ambient air pollutants on the health and lung function of primary school children. The results showed a significant relationship with measured lung function decline in the exposure environment of PM2.5 and PM10. Knibbs et al. [42] studied the children’s health from 7-year-old to 11-year-old in 12 cities in Australia, using satellite land-use regression (LUR) models to estimate NO_2_ concentrations in schools and households for each child. Among the 2630 children, the prevalence of asthma was currently 14.9%. According to estimates, there was an impact between exposure to outdoor NO_2_ and adverse respiratory health on the children in Australia. Roberts et al. [43] explored the problems between air pollutants in urban areas and mental health in childhood and adolescence and found that children under the age of 12 years old exposed to air pollution were not significantly associated with mental health problems. Zaman et al. [44] explored the relationship between energy consumption, environment, health, and its impact on the economic growth of BRICS countries in BRICS countries (Brazil, Russia, India, China and South Africa) during 1975. The results showed that the environment variables have had a detrimental effect on the economic growth of BRICS countries, while energy had significantly increased the economic growth of countries, and it had also been found that health expenditures and infrastructure need to be appropriate for health issues related to fertility and mortality in BRICS countries.

The studies on the energy and environmental efficiency focus on the many objects, such as fossil fuel consumption, CO_2_, SO_2_, and NO_X_ emissions in some countries or in various provinces/states in some one countries. Their research yielded essentially the same results that energy and environmental efficiencies is low, especially for developing area. The studies on the energy and environmental efficiency in EU countries focus on the efficiency of nuclear energy, the energy efficiency of European livestock industry, the efficiency differences among EU countries (Gomez-Calvet; Dumana and Kasman), the GHG emissions (El) and the agricultural efficiency. We can learn from their research that the first 15 countries in EU had greater efficiency and energy and environmental efficiency, still have potential to reduce carbon dioxide emissions and so on to improve the energy and environmental efficiency in EU. The studies on the impact of exposure to environmental pollution on human health mainly focus on the developing countries. For example, China and BRICS countries. Studies have shown that increased PM2.5, PM10, CO_2_, and SO_2_ emissions can significantly harm population health.

Table 1 outlines the abovementioned main research topics: energy consumption, environmental pollution, and human health. Although some traditional DEA methods have been employed on above research areas, few studies have explored their relationship among energy, environment, and health efficiency into one model. Thus, this article employed TMDN-DEA model to comprehensively explore energy and health efficiency of EU countries and non-EU countries.

Based on the above literature analysis, this paper makes the following research hypotheses:

**Hypothesis** **1.** *The average overall efficiency of EU countries is higher than that of non-EU countries.*

**Hypothesis** **2.** *The overall energy efficiency of EU countries is higher than that of non-EU countries.*

**Hypothesis** **3.** *The overall health efficiency of EU countries is higher than that of non-EU countries.*

**Hypothesis** **4.** *In each of the energy efficiencies, EU countries are higher than non-EU countries.*

**Hypothesis** **5.** *In each of the health efficiencies, EU countries are higher than non-EU countries.*

## 3. Research Method 

We used TMDN-DEA model to analyze the energy and health efficiencies. TMDN-DEA model in this paper is developed based on SBM dynamic DEA with meta-frontier (MF) and dynamic group boundary model including two stages.

### 3.1. SBM Dynamic DEA 

Farrell [45] measured the level of productivity of a decision-making unit by the concept of a boundary production function, which connected the most efficient production points into production boundaries, and the gap between any real production point and production boundary represented the inefficiency of the production point. Based on the concept of “boundary,” Charnes et al. [46] put forward the CCR data envelopment analysis model, and Banker et al. [47] extended his hypothesis on scale returns and proposed the BCC model. Since the CCR model and the BCC model measured the radial efficiency, the two models assumed that the inputs and outputs can be adjusted in equal proportions (increase or decrease), and this assumption cannot be applied to some cases. In 2001, Tone proposed the slacks-based measure (SBM) model to measure the slack between the input and output items, and used non-radial estimation method to present SBM efficiency with an efficiency value between 0 and 1. In addition to the CCR, BCC, and SBM models, other scholars also developed the data envelopment analysis. The traditional DEA model converts the efficiency between the two variables through input and output projects, and the conversion process is identified as a “black box.” Färe, Grosskopf, and Whittaker [48] proposed network data envelopment analysis (Network DEA) to apply sub-production technology to explore the impact of input allocation and intermediate wealth on the production process. 

Tone and Tsutsui [49] proposed a weighted slack-based measures network data envelopment analysis model, with the linkage among the departments of the decision-making unit as the basis for the analysis of the Network DEA model. The Network DEA model improved the traditional DEA’s failure to analyze the performance of each department. Tone and Tsutsui [50] extended the SBM model to a dynamic analysis of the slacks-based measure and proposed weighted slack-based measures (Dynamic Network DEA) data envelopment analysis mode, using the linkage between the various departments of the decision-making unit as the basis for the analysis of the Network DEA model, and regarded each department as Sub-DMU (Sub-Decision Making Unit), carry-over activities as linkages, and carry-over activities can be divided into four categories: (1) desirable (good), (2) unwanted (bad), (3) discretionary (changeable), (4) non-discretionary (non-changeable).

### 3.2. The Modified Dynamic Network Model

Since this study considers undesirable output and regional differences in the dynamic network SBM model, we can modify Tone and Tsutsui’s [51] dynamic network model and O’Donnell et al. [52] meta-frontier model to be the modified as meta-frontier dynamic network model. The modified meta-frontier dynamic network model is presented as follows.

Suppose there are n number of DMUs(j=1,…,n), with each having k divisions (k=1,…,K), and T time periods (t=1,…,T). Each of the DMUs has an input and output at time period t and a carryover (link) to the next t+1 time period.

Set $m_{k}$ and $r_{k}$ to represent the input and output in each division K, with (k,h)i representing divisions k to h; $L_{hk}^{}$ being the k and h division set; the input and output, links and carryover definitions are outlined in the following.

Inputs and Outputs

$X_{ijk}^{t}\in R_{+}^{}$$\left(i=1,\ldots ,m_{k}^{};\;j=1,\ldots ,n;K=1\ldots ,K;t=1,\ldots ,T\right)$: refers to input i at time period t for $DMU_{j}$ division k

$y_{rjk}^{t}\in R_{+}^{}$$\left(r=1,\ldots ,r_{k}^{};\;j=1,\ldots ,n;K=1\ldots ,K;t=1,\ldots ,T\right)$: refers to output r in time period t for $DMU_{j}$ division k; if part of the output is not ideal, it is considered an input for the division.

Links

$Z_{j(kh)t}^{t}\in R_{+}^{}$$(j=1;\ldots ;n;l=1;..;L_{hk}^{};t=1;\ldots ;T)0$: refers to the period t links from $DMU_{j}$ division k to division h, with $L_{hk}^{}$ being the number of k to h links.

Z^t^_j(kh)t_ ∈R_+_(j =1; …; *n*; l = 1; …; L_kh_; t = 1; …; T)

Carryovers

$Z_{jkl}^{\left(t,t+1\right)}\in R_{+}^{}$$\left(j=1,\ldots ,n;l=1,..,L_{k}^{};k=1,\ldots k,t=1,\ldots ,T-1\right)$: refers to the carryover of t to the t+1 period from $DMU_{j}$ division k to division h, with $L_{k}^{}$ being the number of carryover items in division k.

### 3.3. Meta-Frontier (MF)

Assuming that all manufacturers (*N*) are composed of decision units of g groups (*N* = *N_1_* + *N_2_* + ….+ *N_g_*) because of different management types, environments, and resources, and x_ij_ and y_rj_ represent respectively the i-th input (i = 1, 2, …, m), the r-th final output r (r = 1, 2, …, s) in the j-th unit (j = 1, 2, …, N). Under the mate boundary, the decision unit k can choose the final output weight that is most favorable to its maximum value, so the efficiency of the decision unit k under the common boundary can be solved by the following linear programming procedure.

$$\rho^{*}=min\frac{\frac{1}{T}\overset{T}{\underset{t=1}{\sum }}W^{t}\left[1-\frac{1}{m+ninput}\left[\overset{G}{\underset{g=1}{\sum }}\overset{m}{\underset{i=1}{\sum }}\frac{S_{it}^{-}}{X_{iot}}+\overset{G}{\underset{g=1}{\sum }}\overset{ninput}{\underset{r=1}{\sum }}\frac{S_{rt}^{input}}{Z_{rot}^{input}}\right]\right]}{\frac{1}{T}\overset{T}{\underset{t=1}{\sum }}W^{t}\left[1+\frac{1}{S_{1}+S_{2}}\left[\overset{G}{\underset{g=1}{\sum }}\overset{S1}{\underset{l=1}{\sum }}\frac{S_{jt}^{+g}}{y_{lot}^{g}}+\overset{G}{\underset{g=1}{\sum }}\overset{S2}{\underset{l=1}{\sum }}\frac{S_{jt}^{-b}}{y_{lob}^{b}}\right]\right]}$$

(1) $$s.t.\;\overset{G}{\underset{g=1}{\sum }}\overset{n}{\underset{\partial =1}{\sum }}Z_{ijtg}\lambda_{jg}^{t}=\overset{G}{\underset{g=1}{\sum }}\overset{n}{\underset{\partial =1}{\sum }}Z_{ijtg}\lambda_{jg}^{t+1}\;\left(vi|t=1\cdots i-1\right)$$

Equation (1) represents the connection equation between term *t* and *t* + 1 

$$X_{iot}=\overset{G}{\underset{g=1}{\sum }}\overset{n}{\underset{\partial =1}{\sum }}X_{ijtg}\lambda_{jg}^{t}+S_{it}\;\left(i=1\cdots m,t=1\cdots i\right)$$

$$y_{\mathrm{lot}}=\sum_{g=1}^{G}\sum_{l=1}^{s1}y_{lot}^{+g}\lambda_{j}^{t}-s_{\mathrm{lt}}^{+g}\left(l=1,\ldots ,s1;t=1,\ldots ,T\right)$$

$$y_{\mathrm{lot}}=\overset{G}{\underset{g=1}{\sum }}\overset{s2}{\underset{l=1}{\sum }}y_{lot}^{-b}\lambda_{j}^{t}-s_{\mathrm{lt}}^{+b}\left(l=1,\ldots ,s2;t=1,\ldots ,T\right)$$

$$Z_{iot}^{good}=\overset{G}{\underset{g=1}{\sum }}\overset{n}{\underset{\partial =1}{\sum }}Z_{ijtg}^{good}\lambda_{jg}^{t}-S_{it}^{t}\;\left(i=1\cdots ngood;t=1\cdots i\right)$$

$$\overset{G}{\underset{g=1}{\sum }}\overset{n}{\underset{\partial =1}{\sum }}\lambda_{jg}^{t}=1\left(t=1\cdots i\right)$$

(2) $$\lambda_{jg}^{t}\geq 0,S_{it}^{-}\geq 0,S_{it}^{+}\geq 0,S_{it}^{good}\geq 0$$

Therefore, we can know the overall technical efficiency (OTE) value of all DMUs under the common boundary model with Equation (2).

### 3.4. Dynamic Group Boundary Model

Assuming that the manufacturer is divided into g groups of decision units, the DMU under each group boundary will choose the most favorable final output weight. Therefore, the efficiency of the DMU under the group boundary will be solved by the following equation:

(3) $$\theta_{0}^{*}=min\frac{\frac{1}{T}\overset{T}{\underset{t=1}{\sum }}W^{t}\left[1-\frac{1}{m+ninput}\left[\overset{m}{\underset{i=1}{\sum }}\frac{S_{it}^{-}}{X_{iot}}+\overset{ninput}{\underset{r=1}{\sum }}\frac{S_{rt}^{input}}{Z_{rot}^{input}}\right]\right]}{\frac{1}{T}\overset{T}{\underset{t=1}{\sum }}W^{t}\left[1+\frac{1}{S_{1}+S_{2}}\left[\overset{S1}{\underset{l=1}{\sum }}\frac{S_{jt}^{+g}}{y_{lot}^{g}}+\overset{S2}{\underset{l=1}{\sum }}\frac{S_{jt}^{-b}}{y_{lob}^{b}}\right]\right]}$$

$$\mathrm{St}\;\sum_{j=1}^{n}z_{ijt}^{\alpha }\lambda_{j}^{t}=\sum_{j=1}^{n}z_{ijt}^{\alpha }\lambda_{j}^{t+1}\left(\forall i;t=1,\ldots ,T-1\right)$$

$$x_{iot}=\sum_{j=1}^{n}x_{ijt}\lambda_{j}^{t}+s_{it}^{-}\;\left(i=1,\ldots ,m;t=1,\ldots ,T\right)$$

$$y_{\mathrm{lot}}=\sum_{l=1}^{s1}y_{lot}^{+g}\lambda_{j}^{t}-s_{\mathrm{lt}}^{+g}\left(l=1,\ldots ,s1;t=1,\ldots ,T\right)$$

$$y_{\mathrm{lot}}=\sum_{l=1}^{s2}y_{lot}^{-b}\lambda_{j}^{t}-s_{\mathrm{lt}}^{-b}\;\left(l=1,\ldots ,s2;t=1,\ldots ,T\right)$$

$$z_{iot}^{good}=\sum_{j=1}^{n}z_{iot}^{good}\lambda_{j}^{t}-s_{it}^{good}\;\left(i=1,\ldots ,ngood;t=1,\ldots ,T\right)$$

$$\sum_{j=1}^{n}\lambda_{j}^{t}\;=1\;\left(t=1,\ldots ,T\right)$$

$$\lambda_{j}^{t}\geq 0,s_{\mathrm{it}}^{-}\geq 0,{\;s}_{\mathrm{it}}^{+}\geq 0,s_{\mathrm{it}}^{\mathrm{good}}\geq 0$$

### 3.5. Technology Gap Ratio (TGR)

Since the meta-frontier model contains g groups, the technical efficiency of the meta-frontier (MFE) will be less than the technical efficiency of the group frontier (GFE). The ratio value, called the technology gap ratio (TGR), is shown as:

(4) $$\mathrm{TGR}=\frac{MFE}{GFE}$$

### 3.6. The Efficiency of Input and Output

We follow Hu and Wang’s [53] total-factor energy efficiency index to overcome any possible bias in the traditional energy efficiency indicator. There are eleven key features of this present study: labor efficiency, non-renewable energy efficiency, renewable energy efficiency, GDP efficiency, health expenditure efficiency, tuberculosis rate efficiency, mortality rate of children efficiency, mortality rate of adult efficiency, survival rate of 65 years old, CO_2_ efficiency, and PM2.5 efficiency. In our study, “I” represents area and “t” represents time. The eleven efficiency models are defined in the following:

(5) $$\mathrm{Labor}\;\mathrm{efficiency}\;=\;\frac{\mathrm{Target}\;\mathrm{Labor}\;\mathrm{input}\;\left(i,t\right)}{\mathrm{Actual}\;\mathrm{Labor}\;\mathrm{input}\;\left(i,t\right)}$$

(6) $$\mathrm{Non}-\mathrm{renewable}\;\mathrm{Energy}\;\mathrm{efficiency}\;=\;\frac{\mathrm{Target}\;\mathrm{non}-\mathrm{renewable}\;\mathrm{energy}\;\mathrm{input}\;\left(i,t\right)}{\mathrm{Actual}\;\mathrm{non}-\mathrm{renewable}\;\mathrm{energy}\;\mathrm{input}\;\left(i,t\right)}$$

(7) $$\mathrm{Renewable}\;\mathrm{Energy}\;\mathrm{efficiency}\;=\;\frac{\mathrm{Target}\;\mathrm{renewable}\;\mathrm{energy}\;\mathrm{input}\;\left(i,t\right)}{\mathrm{Actual}\;\mathrm{renewable}\;\mathrm{energy}\;\mathrm{input}\;\left(i,t\right)}$$

(8) $$\mathrm{GDP}\;\mathrm{efficiency}\;=\frac{\mathrm{Actual}\;\mathrm{GDP}\;\mathrm{desirable}\;\mathrm{output}\;\left(i,t\right)}{\mathrm{Target}\;\mathrm{GDP}\;\mathrm{desirable}\;\mathrm{output}\;\left(i,t\right)}$$

(9) $${\mathrm{CO}}_{2}\;\mathrm{efficiency}\;=\frac{\mathrm{Target}\;{\mathrm{CO}}_{2}\;\mathrm{Undesirable}\;\mathrm{output}\;\left(i,t\right)}{\mathrm{Actual}\;{\mathrm{CO}}_{2}\;\mathrm{Undesirable}\;\mathrm{output}\;\left(i,t\right)}$$

(10) $$\mathrm{PM}2.5\;\mathrm{efficiency}\;=\frac{\mathrm{Target}\;\mathrm{Pm}2.5\;\mathrm{Undesirable}\;\mathrm{output}\;\left(i,t\right)}{\mathrm{Actual}\;\mathrm{Pm}2.5\;\mathrm{Undesirable}\;\mathrm{output}\;\left(i,t\right)}$$

(11) $$\mathrm{Health}\;\mathrm{Expenditure}\;\mathrm{efficiency}=\;\frac{\mathrm{Target}\;\mathrm{Health}\;\mathrm{Expenditure}\;\mathrm{input}\;\left(i,t\right)}{\mathrm{Actual}\;\mathrm{Health}\;\mathrm{Expenditure}\;\mathrm{input}\;\left(i,t\right)}$$

(12) $$\mathrm{Tuberculosis}\;\mathrm{rate}\;\mathrm{efficiency}\;=\frac{\mathrm{Target}\;\mathrm{Tuberculosis}\;\mathrm{rate}\;\mathrm{output}\;\left(i,t\right)}{\mathrm{Actual}\;\mathrm{Tuberculosis}\;\mathrm{rate}\;\mathrm{output}\;\left(i,t\right)}$$

(13) $$\mathrm{Mortality}\;\mathrm{rate}\;\mathrm{of}\;\mathrm{children}\;\mathrm{efficiency}\;=\frac{\mathrm{Target}\;\mathrm{Mortality}\;\mathrm{rate}\;\mathrm{of}\;\mathrm{children}\;\mathrm{output}\;\left(i,t\right)}{\mathrm{Actual}\;\mathrm{Mortality}\;\mathrm{rate}\;\mathrm{of}\;\mathrm{children}\;\mathrm{output}\;\left(i,t\right)}$$

(14) $$\mathrm{Mortality}\;\mathrm{rate}\;\mathrm{of}\;\mathrm{adult}\;\mathrm{efficiency}\;=\frac{\mathrm{Target}\;\mathrm{Mortality}\;\mathrm{rate}\;\mathrm{of}\;\mathrm{adult}\;\mathrm{output}\;\left(i,t\right)}{\mathrm{Actual}\;\mathrm{Mortality}\;\mathrm{rate}\;\mathrm{of}\;\mathrm{adult}\;\mathrm{output}\;\left(i,t\right)}$$

(15) $$\mathrm{Survival}\;\mathrm{rate}\;\mathrm{of}\;65\;\mathrm{years}\;\mathrm{old}\;\mathrm{efficiency}\;=\frac{\mathrm{Actual}\;\mathrm{Survival}\;\mathrm{rate}\;\mathrm{of}\;65\;\mathrm{years}\;\mathrm{old}\;\mathrm{desirable}\;\mathrm{output}\left(i,t\right)}{\mathrm{Target}\;\mathrm{Survival}\;\mathrm{rate}\;\mathrm{of}\;65\;\mathrm{years}\;\mathrm{old}\;\mathrm{desirable}\;\mathrm{output}\left(i,t\right)}$$

If the target labor, non-renewable energy efficiency, renewable energy efficiency, and health expenditure input equals the actual input, then the labor, non-renewable energy efficiency, renewable energy efficiency, and health expenditure efficiencies equal 1, indicating overall efficiency. If the target labor, non-renewable energy efficiency, renewable energy efficiency, and health expenditure input is less than the actual input, then the labor, non-renewable energy efficiency, renewable energy efficiency, and health expenditure efficiencies are less than 1, indicating overall inefficiency.

If the target tuberculosis rate efficiency, mortality rate of children efficiency, mortality rate of adult efficiency, CO_2_, and PM2.5 undesirable outputs equal the actual undesirable outputs, then tuberculosis rate efficiency, mortality rate of children efficiency, mortality rate of adult efficiency, CO_2_, and PM2.5 efficiencies equal 1, indicating overall efficiency. If the target tuberculosis rate efficiency, mortality rate of children efficiency, mortality rate of adult efficiency, CO_2_, and AQI undesirable outputs are less than the actual undesirable outputs, then the tuberculosis rate efficiency, mortality rate of children efficiency, mortality rate of adult efficiency, CO_2_, and PM2.5 efficiencies are less than 1, indicating overall inefficiency. 

If the target GDP and survival rate of 65-years-old desirable output is equal to the actual GDP and survival rate of 65-years-old desirable output, then the GDP and survival rate of 65-years-old efficiency equals 1, indicating overall efficiency. If the actual GDP and survival rate of 65-years-old desirable output is less than the target GDP desirable output, then the GDP and survival rate of 65-years-old efficiency is less than 1, indicating overall inefficiency. 

**Figure 2:**
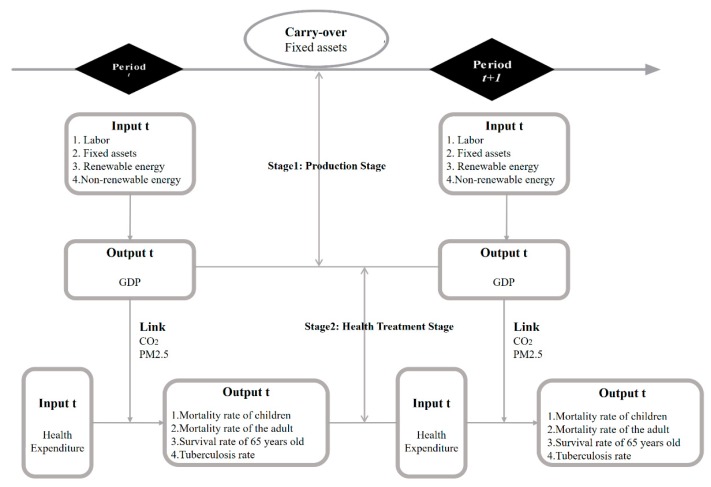
Two-stage meta dynamic network data envelopment analysis (DEA) model.

Figure 2 reveals the framework of the modified meta dynamic network DEA model of inter-temporal efficiency measurement and variables in this study.

## 4. Empirical Study 

### 4.1. Data Sources and Description

This study compares the energy efficiency and healthy efficiency in EU and non-EU countries from 2010 to 2014. The diseases data are extracted from Global Tuberculosis Control Report (World Health Organization) [54] and the others are from World Development Indicators of the World Bank. Now, there are 28 EU member countries. Based on the data availability, we choose 53 countries as non-EU countries from the other countries. 

In our study, the variables in each stage are showed in the Table 2. The first stage is production stage including three input variables and one output variables. The second stage is the health treatment stage including one input variable and four output variables. 

*The first Stage: Production Stage*

Input Variables:

Labor: The numbers of employees in each country by the end of each year. Unit: Million person. 

Renewable energy: Renewable energy consumption in each country every year. Unit: Mega Joule.

Non-renewable energy: Non-renewable energy consumption in each country each year. Unit: Mega Joule.

Output Variables:

GDP (desirable output): GDP in each country each year. Unit: billion dollars at current price.

*The Second Stage: the Health Treatment Stage*

Input Variables:

Healthy Expenditure: Total annual health expenditure in each country. Unit: billion dollars.

Output Variables:

Mortality rate of children (undesirable output): Mortality rate of children that is less than 5 years old in each country each year. Unit: percent.

Mortality rate of the aged (undesirable output): Mortality rate of the aged that is more than 65 years old in each country each year. Unit: percent.

Survival rate (desirable output): Survival rate is that of 65 years old in each country each year. Unit: percent.

Tuberculosis rate (undesirable output): Excessive content of CO_2_ and PM2.5 in the air will reduce people’s immunity to pulmonary tuberculosis, thus increasing the incidence of pulmonary tuberculosis. Tuberculosis rate is that in each country each year. Unit: ‱.

*Link Production Stage and the Health Treatment Stage Variables*

CO_2_: CO_2_ emissions in each country each year. Unit: million ton.

PM2.5: The content of PM2.5 in the air in each country each year. Unit: Micrograms per cubic meter.

*Carry Over Production Stage and the Health Treatment Stage Variable*

Fixed assets: Capital stock of each country is calculated by fixed assets investment in each country by the end of each year. Unit: Billion dollars.

### 4.2. Input and Output Variables Statistical Analysis

Before the empirical study, this paper has carried out isotonicity test to justify the selection of variables. Table 3 shows a statistical table of the overall input and output variables of EU countries. As we show, they are labor by million persons, capital fixed assets by billion dollars, renewable energy by mega joule, non-renewable energy by mega joule, GDP by billion dollars, CO2 by million ton, PM2.5 by micrograms per cubic meter, health expenditure by billion dollars, tuberculosis rate per ten thousand, mortality rate of children by percent, mortality rate of adults by percent and survival rate of 65 years old by percent. The average values of PM2.5, CO2, non-renewable energy, tuberculosis rate, and mortality rate of children and adults and survival rate of 65 years old decreased obviously from 2010 to 2014. The average values of labor, capital, GDP, and health expenditure are not changed too much.

The minimum values of most variables are nearly 0 and they are not very clear in the picture. The minimum values of PM2.5, mortality rate of children and adults declined from 2010 to 2014. The minimum values of labor and survival rate of 65 years old were not change too much from 2010 to 2014.

The maximum values of labor and survival rate of 65 years old were not changed too much from 2010 to 2014. The maximum and average values of CO2, non-renewable energy, tuberculosis rate, mortality rate of children and adults declined from 2010 to 2014. The maximum and average values of capital, GDP, renewable energy, and health expenditure increased from 2010 to 2014.

Table 4 shows a statistical table of the overall input and output variables of non-EU countries. The minimum values of most variables are nearly 0. 

The average values of labor, capital, renewable energy, non-renewable energy, and GDP were not change too much from 2010 to 2014. The average values of PM2.5, survival rate of 65 years old, tuberculosis rate, mortality rate of children and adults declined from 2010 to 2014. The average values of health expenditure increased from 2010 to 2014.

The minimum values of PM2.5 decreased a little and that of mortality rate of children and adults were not change too much from 2010 to 2014. But the minimum value of survival rate of 65 years old increased significantly from 2010 to 2014.

The maximum values of tuberculosis rate, mortality rate of children and adults declined from 2010 to 2014. The maximum values of CO_2_, non-renewable energy, capital, GDP, renewable energy, and health expenditure increased from 2010 to 2014. The maximum values of labor and survival rate of 65 years old were not change from 2010 to 2014.

### 4.3. Total Annual Efficiency Scores

The overall efficiencies in each EU country from 2010 to 2014 are showed in the Table 5. An overall efficiency of 1 in all four years was achieved by Malta. Cyprus and Sweden’s efficiencies were 1 in 2012 and 2013. Luxembourg’s efficiency was 1 in 2010, but that declined below 0.7 from 2011.Therefore, there are many other EU countries that need to improvement the efficiency scores. 

Bulgaria, Hungary, Poland, and Romania’s efficiencies of each year were below 0.2 from 2010 to 2014 and Czech’s overall efficiency was also below 0.2, and the efficiencies of Austria, Croatia, Latvia, Lithuania, and Slovak were between 0.2 to 0.3. These countries are all local in the east Europe. Except Malta, Cyprus, and Sweden; there are Finland, Greece, Italy, Luxembourg, Netherlands, and Sweden where efficiencies were over 0.5. Therefore, there were 9 countries where efficiencies were over 0.5 and 10 countries where efficiencies were below 0.3 in EU countries of 28.

In the non-EU countries of 53, there are 6 countries where overall efficiencies were 1 in all four years, as showed in the Table 6. These countries are Brunei, Japan, Iceland, Saudi Arabia, Singapore, and United States. There are four years that United Arab Emirates and Korea’s efficiencies were 1 in five years. Australia, Mongolia, and Norway’s efficiencies were 1 in 2010, but those declined below 0.6 in 2014. Switzerland’s efficiency was 1 in 2012 to 2013, but those overall efficiencies were below 0.6 in other years.

Australia, Canada, Korea, New Zealand, Switzerland, United Arab Emirates total efficiencies were above 0.6 and those countries are developing countries. Albania, Cambodia, Cuba, Nepal, Norway, Iraq, Israel’s total efficiencies were between 0.5 to 0.6. Therefore, there were 19 countries where efficiencies were over 0.5 in non-EU countries of 53.

India, Vietnam, South Africa, and Ukraine’s efficiencies were below 0.1 from 2010 to 2014. Those countries’ efficiencies were the lowest. Brazil, Bangladesh, Belarus, Chile, China, Colombia, Iran, Morocco, Kazakhstan, Kenya, Malaysia, Mexico, Peru, Philippines, Thailand, Tunisia, and Turkey’s efficiencies were below 0.2 in most or all year from 2010 to 2014. Algeria, Argentina, Cameroon, Costa Rica, Pakistan, and Sri Lanka’s total efficiencies were between 0.2 to 0.3. Therefore, there were 27 countries where efficiencies were below 0.3 in non-EU countries of 53.

The overall efficiencies in non-EU countries were worse than those in EU countries. There are half countries where overall efficiencies were below 0.3 but that is one-third of the EU countries. We can also prove this from the average value of overall efficiencies in EU and non-EU countries. The average values of overall efficiencies are higher in EU countries than that in non-EU countries each year from 2010 to 2014. This basically proves the hypothesis:”H1: The average overall efficiency of EU countries is higher than that of non-EU countries.”

### 4.4. Total Average Efficiency Scores Analysis in Each Stage

From the view of each stage, the overall efficiencies of EU and non-EU countries have different performance. Because there are too many countries in EU and non-EU, we use the average values of the overall efficiencies of EU and non-EU countries to compare the difference between EU and non-EU countries. We can see the results in the Table 7, the average overall efficiencies of EU countries are higher than those of non-EU countries each year from 2010 to 2014 in the first stage (production stage). The average overall efficiencies of non-EU countries are below 0.47 in the first stage, but those of EU countries are above 0.61 for each year. Therefore, the energy efficiencies in non-EU countries is lower than EU countries and there are much more spaces to improve the energy efficiencies in non-EU countries. This basically proves the hypothesis:”H2: The overall energy efficiency of EU countries is higher than that of non-EU countries.”

But the average overall efficiencies of EU countries are lower than those of non-EU countries each year from 2010 to 2014 in the second stage (health treatment stage). The average overall efficiencies of EU countries are below 0.27 except 0.3382 in 2010 from 2010 to 2014 in the second stage, but those of EU countries are above 0.34 for each year from 2010 to 2014. Therefore, the healthy efficiencies in EU countries is lower than non-EU countries and there is much more space to improve the healthy efficiencies in EU countries.

Meanwhile, the average overall efficiencies of EU and non-EU countries each year in the second stage are all lower than those in the first stage, especially for EU countries. Thus, there are much more spaces to improve for the healthy efficiencies than for the energy efficiencies in EU countries and non-EU countries. This proves that the hypothesis “H3: The overall health efficiency of EU countries is higher than that of non-EU countries” is not true.

### 4.5. The Technical Efficiency of the Group Frontier for EU and Non-EU countries

We can learn the technical efficiency of the group frontier for EU and non-EU countries from the technology gap ratio (TGR) of EU and non-EU countries from 2010 to 2014, as showed in Table 8. In the non-EU countries of 53, there are 7 countries where TGRs were 1 in all four years. These countries are Brunei, Japan, Iceland, Nigeria, Saudi Arabia, Singapore, and United States. United Arab Emirates and Korea’s efficiencies were 1 in five years. In the EU of 28 countries, there are only 1 country where TGR was 1 in all four years and it is Malta. 

Table 8 showed that the average overall TGRs were higher obviously each year in non-EU countries than those countries in EU. Only the average overall TGR was over 0.7 in 2010 for EU countries, another TGRs were below 0.37 from 2011 to 2014. But TGRs were above 0.85 for non-EU countries each year from 2010 to 2014.

The explanation for this phenomenon is mainly due to the TGRs of EU countries in the second stage was lower significantly than non-EU countries from 2011 to 2014. The TGRs of EU countries in the second stage were all below 0.48, but those of non-EU countries were above 0.84 each year from 2010 to 2014. However, in the first phase, the TGRs of EU countries were higher than those of non-EU countries. Thus, EU countries have a bigger gap between group frontier (GF) and meta-frontier (MF) in the healthy treatment stage, and the TGRs of non-EU countries were very close and high in the production stage and the healthy treatment stages from 2011 to 2014.

### 4.6. The Efficiency of the Input and Output Variables

We can learn the energy efficiencies for the inputs and outputs from the production stage in EU countries and non-EU countries. As the Table 9 showed, the GDP efficiencies were all above 0.92 each year for EU countries and non-EU countries. The non-renewable energy, renewable energy, labor, PM2.5, and CO_2_ efficiencies of EU countries were all higher than ones of non-EU countries from 2010 to 2014. The gap of renewable energy, PM2.5 and CO_2_ efficiencies between EU countries and non-EU countries were more significant. There are much more space for the non-EU countries to improve the energy efficiencies of inputs and outputs. This basically proves the hypothesis: ”H4: In each of the energy efficiencies, EU countries are higher than non-EU countries.”

Meanwhile, the renewable energy efficiencies were obviously higher than the non-renewable energy efficiencies and PM2.5 efficiencies were obviously higher than the CO_2_ efficiencies for EU countries and non-EU countries.

We can learn the health efficiencies for the inputs and outputs from the health treatment stage in EU countries and non-EU countries. From the Table 10, we can see the survival rate efficiencies were all above 0.91 each year for EU countries and non-EU countries and there were 3 years that their values were 1 in all four years in EU countries. The tuberculosis rate efficiencies and mortality rate of children efficiencies of EU countries were all higher than one for non-EU countries from 2010 to 2014. The health expenditure efficiencies and the mortality rate of the adult efficiencies of EU countries were all lower than ones for non-EU countries from 2010 to 2014, except the mortality rate of the adult efficiencies in the 2014. There is much more space for the non-EU countries to improve the tuberculosis rate efficiencies and mortality rate of children efficiencies, and there are much more space for the EU countries to improve the health expenditure efficiencies and the mortality rate of the adult efficiencies. Meanwhile, the mortality rate of children efficiencies was higher than the mortality rate of the adult efficiencies for EU countries and non-EU countries. This proves the hypothesis: ”H5: In each of the health efficiencies, EU countries are higher than non-EU countries” is not true.

Because the health expenditure efficiencies are very low and there is different performance between the children and the adult mortality rate, we analyzed in detail the specific situation of health efficiencies of each EU and non-EU country. The results are showed in the Table 9 and Table 10.

In the EU countries (see Table 11), there are 2 countries where health expenditure efficiencies were 1 in all five years and they are Cyprus and Malta. There are 13 countries where health expenditure efficiencies were below 0.1 in the EU countries. Most of them are high welfare countries, for example, Belgium, Denmark, France, UK, Iceland, Netherlands, Spain, and Portugal.

There are 7 countries where children’s mortality rate efficiencies were 1 in all five years and they are Cyprus, Estonia, Finland, Lithuania, Latvia, Slovenia, and Malta. The children’s mortality rate efficiency in Slovak was at the least and it was 0.5693. Another countries’ children’s mortality rate efficiencies were above 0.62 in EU. 

There are 3 countries where adult’s mortality rate efficiencies were 1 in all five years and they are Cyprus, Italy, and Malta. There are 6 countries where adult’s mortality rate efficiencies were above 0.8 in all five years and they are Bulgaria, Germany, Spain, Luxembourg, Netherlands, and Sweden. The adult’s mortality rate efficiencies in Estonia, Hungary, and Lithuania were lower than the ones of other countries and they were 0.3787,0.3218, 0.2700 respectively.

In the non-EU countries (see Table 12), there are 9 countries where health expenditure efficiencies were 1 in all five years and they are Albania, United Arab, Brunei, Iceland, Japan, Nepal, Saudi Arabia, Singapore, and USA. There are 11 countries where health expenditure efficiencies were below 0.1 in the non-EU countries. They are Argentina, Chile, Colombia, India, Mexico, Peru, Thailand, Turkey, Ukraine, Vietnam, and South Africa.

There are 12 countries where the mortality rate of children efficiencies was 1 in all five years and they are Albania, United Arab, Belarus, Brunei, Iceland, Japan, Cambodia, Korea, Nepal, Saudi Arabia, Singapore, and USA. There are still other 8 countries where children’s mortality rate efficiencies were above 0.8. But there are 15 countries where children’s mortality rate efficiencies were below 0.56 in all five years and the least value is 0.2742 in Brazil. But children’s mortality rate efficiencies were all above 0.56 in EU countries each year. 

There are 11 countries where adult’s mortality rate efficiencies were 1 in all five years and they are Albania, United Arab, Brunei, Canada, China, Iceland, Japan, Nepal, Saudi Arabia, Singapore, and USA. There are still other 10 countries where adult’s mortality rate efficiencies were above 0.8 in all five years. There are 5 countries where adult’s mortality rate efficiencies were below 0.4 in all five years and they are Belarus, Botswana, Colombia, Kazakhstan, and Philippines in non-EU countries of 53 and they were 0.2221, 0.3831, 0.3381, 0.2346, 0.3576 respectively. Relatively speaking, there are 3 countries where adult’s mortality rate efficiencies were below 0.4 in EU countries of 28. 

## 5. Conclusions 

This study focuses on the energy efficiencies and health efficiencies in 28 EU countries and 53 non-EU countries from 2010 to 2014. Using a TMDN-DEA model, we calculate the overall efficiencies score and the technology gap ratios of each EU and non-EU countries from 2010 to 2014 in each and total stages. The first stage is production stage and the second stage is health treatment stage. Then we also calculate the efficiencies for the inputs and outputs of the production and health stage in EU countries and non-EU countries, including the non-renewable energy, renewable energy, PM2.5, CO_2_, labor, GDP, tuberculosis rate, children’s mortality rate, adult’s mortality rate, health expenditure efficiencies, and survival rate efficiencies. Finally, we have a generalization of the results of the study. First, the average overall efficiencies in EU countries were higher than in non-EU countries. Second, EU countries have higher energy efficiencies than non-EU countries and non-EU countries have higher health efficiencies than EU countries. Third, the renewable energy efficiencies were higher than the non-renewable energy efficiencies. The detail conclusions from analysis are as follows.

1. Average overall efficiencies each year in EU countries were higher than in non-EU countries from 2010 to 2014. An overall efficiency of 1 in all four years was achieved by Malta which are EU countries and Brunei, Japan, Iceland, Saudi Arabia, Singapore, and United States which are non-EU countries.
2. These countries where average overall efficiencies are lower in EU are all located in the east Europe, for example, Bulgaria, Hungary, Poland, Czech, Romania, Austria, Croatia, Latvia, Lithuania, and Slovak. The countries where average overall efficiencies are lower in non-EU are developing countries. For example, India, Vietnam, South Africa, Ukraine Brazil, Bangladesh, Belarus, Chile, China, Colombia, Iran, Morocco, Kazakhstan, Kenya, Malaysia, Mexico, Peru, Philippines, Thailand, Tunisia, and Turkey.
3. EU countries have higher energy efficiencies than non-EU countries and non-EU countries have higher health efficiencies than EU countries. The average overall efficiencies of EU countries are higher than those of non-EU countries each year from 2010 to 2014 in the first stage (production stage). But the average overall efficiencies of EU countries are lower than those of non-EU countries each year from 2010 to 2014 in the second stage (health treatment stage).
4. There is much more space to improve the healthy efficiencies for the countries in EU. The average overall efficiencies in the second stage are all lower than those in the first stage both for EU countries and for non-EU countries each year, especially it is obviously for EU countries.
5. EU countries have a bigger gap between group frontier (GF) and meta-frontier (MF) in the healthy treatment stage. Although the TGRs of EU countries were higher than the ones of non-EU countries in the first stage, the TGRs of EU countries were too low than the non-EU countries. Thus, the technical efficiency of the group frontier of non-EU countries is higher than EU countries.
6. The renewable energy efficiencies were higher obviously than the non-renewable energy efficiencies and PM2.5 efficiencies were higher obviously than the CO_2_ efficiencies for EU countries and non-EU countries. But there is much more space for the non-EU countries to improve the energy efficiencies of inputs and outputs.
7. There is much more space for the non-EU countries to improve the tuberculosis rate efficiencies and children’s mortality rate efficiencies, and there are much more space for the EU countries to improve the health expenditure efficiencies and the adult’s mortality rate efficiencies.
8. The children’s mortality rate efficiencies were higher than the adult’s mortality rate efficiencies for EU countries and non-EU countries. There are 19 countries where the children’s mortality rate efficiencies were 1, but there are 14 countries where the children’s mortality rate efficiencies were 1 in all five years.
9. The health expenditure efficiencies in the EU countries are obviously lower than those in non-EU countries. In the non-EU, there are 9 countries where health expenditure efficiencies were 1, but there are 2 countries in EU. There are 13 countries where health expenditure efficiencies were below 0.1 in the non-EU countries, but there are 11 countries in EU. Most of countries where the health expenditure efficiencies were below 0.1 in the EU countries are high welfare countries, for example, Belgium, Denmark, France, UK, Iceland, Netherlands, Spain, and Portugal.

In theory, this paper has enough literature to prove the significance and value of our research. First of all, we applicate a modified meta dynamic network model with a production stage to analysis renewable and non-renewable energy efficiency. Second, we have a second health treatment stage to focus on health expenditure and the impact on survival rate of 65 years old, children’s and adults’ mortality rate in EU countries and non-EU countries. Through the conclusion of this paper, this paper puts forward the following practical and positive suggestions for the EU and non-EU countries.

- As we known from conclusions, the non-renewable energy efficiencies are much lower than the renewable energy efficiencies in the EU and non-EU countries. The government management in the EU and non-EU countries should be strengthened to reduce air pollutant and carbon dioxide emissions which are from the consumption of the non-renewable energy. Moreover, EU-ETS should play a greater role. The price of emission right has affected the production decision of enterprises. If enterprises do not take emission reduction measures, they need to bear more cost of emission reduction. This is conducive to the transformation of energy consumption of enterprises to renewable energy.
- Because the efficiencies of renewable energy are higher than ones of non-renewable, so it is more inclined to use renewable energy, especially clean energy in renewable energy, such as solar energy, wind energy, and so on. Further attention should be given to raise energy transformation to the clean energy in renewable energy. At the same time, the efficiency improvement of renewable energy is to improve the efficiency of energy management. The efficiency improvement of energy transmission and distribution management will enhance the utilization efficiency of new energy. EU countries and non-EU countries should encourage the application of new technologies in new energy development and energy management efficiency improvement.
- Because of the differences of the health efficiencies are serious among the non-EU countries and the health efficiencies in developing countries are lower than ones of developed countries. These developing countries should concentrate more on the health expenditure. Health expenditure is the basic social security. Non-EU governments should consider establishing corresponding laws and regulations to protect health expenditure.
- As we known from conclusions, the health expenditure efficiency of EU countries is even much lower than non-EU countries. Meanwhile, most of these EU countries are the high-welfare countries. These high-welfare countries in Western Europe should consider how to make full use of medical resources and improve medical efficiency. The government of high welfare countries should actively encourage new medical technology to improve health efficiency while ensuring health expenditure.
- The health efficiencies are low in the EU and non-EU countries, health expenditure efficiency should be improved. Especially in EU countries, there are too much space to reduce their adult’s mortality rate. The prompt of health efficiency not only depends on the improvement of technology, but also the management efficiency of government in health expenditure.

## Figures and Tables

**Table 1:** Comparison of previous studies and this study.

Previous Studies	This Study
Research on the energy consumption and environment efficiency [3,4,5,6,7,8,9,10,11,12,13,14,15,16,17,18].	Application of a modified meta dynamic network model with a production stage to analyze renewable and non-renewable energy efficiency, and a second health treatment stage focused on health expenditure and the impact on survival rate of 65 years old, children and adult mortality rate in EU countries and non-EU countries.
Research on the EU countries’ energy and environmental issues [19,20,21,22,23,24,25,26,27,28,29].	
Research on the relationship between environmental pollution on human health [30,31,32,33,34,35,36,37,38,39,40,41,42,43,44].	

**Table 2:** Input and output variables.

Stage	Input Variables	Output Variables	Link	Carry Over
Stage 1	Labor by million persons	GDP by billion dollars	CO_2_ by million ton	Fixed assets by billion dollars
	Renewable energy by mega joule		PM2.5 by micrograms per cubic meter	
	Non-renewable energy by mega joule			
Stage 2	Health Expenditure by billion dollars	Mortality rate of children (less than 5 years old) by percent		
		Mortality rate of the adult (from 15 to 65 years old) by percent		
		Survival rate of 65 years old by percent		
		Tuberculosis rate by ‱		

**Table 3:** Statistics of input and output variables of EU countries.

Item	Year	Labor	Capital	Re Energy	Non-Re Energy	GDP	CO_2_	PM2.5	Health Exp ^1^	Tub Rate ^2^	Mor.Ch ^3^	Mor.Ad ^4^	Sur Rate ^5^
Average	2010	7.87	121.74	213.37	1433.03	607.49	132.49	15.83	46.59	0.02	0.51	19.41	73.00
	2011	7.85	132.24	207.78	1350.08	656.24	127.43	16.02	49.87	0.02	0.49	18.88	62.35
	2012	7.83	121.82	226.00	1333.93	618.46	124.79	14.78	47.22	0.02	0.47	17.78	52.36
	2013	7.82	124.15	238.67	1320.52	644.75	122.20	14.29	50.41	0.02	0.46	17.29	48.24
	2014	7.91	129.14	241.26	1246.13	666.76	115.78	13.81	52.23	0.02	0.45	16.74	46.85
Max	2010	39.09	664.13	889.17	7748.99	3417.09	758.86	27.18	313.45	0.11	1.15	38.23	89.36
	2011	39.27	761.54	935.66	7275.93	3757.70	732.50	26.37	335.06	0.10	1.13	36.90	90.14
	2012	39.56	712.77	997.31	7303.14	3543.98	739.86	24.26	317.21	0.09	1.11	35.95	90.14
	2013	40.00	739.22	1030.49	7493.64	3752.51	757.31	22.93	343.77	0.09	1.06	35.74	90.14
	2014	40.34	779.52	1087.82	7043.06	3898.73	719.88	22.21	360.58	0.09	1.00	33.48	90.14
Min	2010	0.16	1.87	0.23	16.62	8.74	2.56	7.19	0.46	0.00	0.30	11.32	7.42
	2011	0.17	1.71	0.33	15.97	9.50	2.54	7.28	0.52	0.00	0.29	11.20	7.42
	2012	0.17	1.67	0.44	16.62	9.21	2.68	6.60	0.52	0.01	0.28	10.92	7.42
	2013	0.18	1.77	0.45	17.16	10.15	2.34	6.28	0.61	0.01	0.26	10.58	7.42
	2014	0.19	1.94	0.71	17.48	11.28	2.35	6.47	0.65	0.00	0.25	10.27	7.42
St. Dev ^6^	2010	10.10	177.94	243.93	1951.48	905.40	177.88	4.84	76.20	0.02	0.21	7.50	21.96
	2011	10.12	196.23	234.00	1820.57	981.83	170.35	4.87	81.36	0.02	0.20	7.25	29.92
	2012	10.15	182.99	258.56	1831.23	933.66	171.07	4.52	77.48	0.02	0.19	6.87	32.89
	2013	10.20	187.71	271.71	1843.31	975.36	171.28	4.29	83.90	0.02	0.18	6.71	32.06
	2014	10.31	196.39	273.61	1722.50	1018.17	160.71	4.11	88.22	0.02	0.17	6.57	31.59

^1^ Healthy expenditure; ^2^ Tuberculosis rate; ^3^ Mortality rate of children; ^4^ Mortality rate of the aged; ^5^ Survival rate; ^6^ Standard deviation.

**Table 4:** Statistics of input and output variables of non-EU countries.

Item	Year	Labor	Capital	Re Energy	Non-Re Energy	GDP	CO_2_	PM2.5	Health Exp ^1^	Tub Rate ^2^	Mor.Ch ^3^	Mor.Ad ^4^	Sur Rate ^5^
Average	2010	42.21	209.14	726.94	3876.00	848.65	489.35	32.36	45.94	0.13	2.43	29.79	72.14
	2011	42.61	240.58	739.55	4001.06	953.12	511.98	32.38	51.91	0.13	2.32	29.06	71.26
	2012	42.99	259.19	759.33	4042.52	997.89	523.19	31.50	54.57	0.12	2.22	28.15	67.90
	2013	43.46	270.43	778.22	4130.98	1021.12	529.45	30.84	55.19	0.12	2.12	27.66	63.76
	2014	43.92	280.22	794.10	4201.18	1044.83	537.27	29.67	74.50	0.12	2.04	27.17	57.82
Max	2010	744.86	2756.06	7871.45	53,328.08	14,992.05	8776.04	100.78	1194.31	0.95	12.96	89.88	89.36
	2011	747.79	3399.69	7655.96	57,803.12	15,542.58	9733.54	100.77	1233.85	0.92	12.47	84.98	90.14
	2012	749.38	3874.99	8069.99	59,356.54	16,197.01	10,028.57	96.96	1284.60	0.89	11.99	80.09	90.14
	2013	750.81	4372.71	8275.65	61,630.89	16,784.85	10,258.01	95.31	1339.61	0.86	11.56	76.82	90.14
	2014	751.92	4721.38	8744.47	62,791.84	17,521.75	10,291.93	98.12	2353.21	0.82	11.16	73.55	90.14
Min	2010	0.17	1.33	0.01	25.87	4.79	1.96	7.15	0.14	0.00	0.27	10.69	36.84
	2011	0.17	1.47	0.01	24.83	6.20	1.88	7.37	0.16	0.00	0.26	10.11	7.42
	2012	0.18	2.07	0.01	24.64	6.61	1.80	6.84	0.17	0.00	0.25	10.12	7.42
	2013	0.18	2.17	0.01	26.81	7.34	1.90	6.71	0.17	0.00	0.24	10.57	7.42
	2014	0.19	2.43	0.01	27.00	7.47	1.98	6.18	0.19	0.00	0.23	10.00	7.42
St. Dev ^6^	2010	116.63	540.26	1645.88	10,237.27	2278.83	1394.72	21.79	171.07	0.18	2.64	17.78	13.22
	2011	116.97	621.21	1662.53	10,600.66	2436.40	1500.75	21.75	182.13	0.18	2.52	17.00	22.99
	2012	117.19	688.57	1713.30	10,690.19	2562.51	1529.36	21.27	189.64	0.17	2.42	16.29	27.08
	2013	117.88	745.37	1753.42	11,000.86	2653.38	1557.78	20.98	192.92	0.17	2.32	15.76	29.19
	2014	118.62	798.57	1805.75	11,223.53	2780.17	1569.61	20.52	324.34	0.16	2.24	15.33	30.93

^1^ Healthy expenditure; ^2^ Tuberculosis rate; ^3^ Mortality rate of children; ^4^ Mortality rate of the aged; ^5^ Survival rate; ^6^ Standard deviation.

**Table 5:** Overall efficiency by EU countries from 2010 to 2014.

No.	DMU 1	2010	2011	2012	2013	2014	Annual Average	No.	DMU	2010	2011	2012	2013	2014	Annual Average
1	Austria	0.3254	0.2855	0.2925	0.3035	0.2936	0.2687	16	Latvia	0.2279	0.2592	0.2615	0.2761	0.2983	0.2635
2	Belgium	0.4094	0.4124	0.3752	0.3875	0.3742	0.3498	17	Lithuania	0.2192	0.3063	0.2422	0.2630	0.2331	0.2372
3	Bulgaria	0.1513	0.1713	0.1705	0.1734	0.1572	0.1620	18	Luxembourg	1	0.6521	0.6343	0.6278	0.6363	0.6866
4	Croatia	0.2178	0.2105	0.2185	0.2356	0.2078	0.2067	19	Malta	1	1	1	1	1	1
5	Cyprus	0.8020	0.7983	1	1	0.9206	0.9023	20	Netherlands	0.5992	0.5262	0.5314	0.5294	0.5310	0.5184
6	Czech	0.2099	0.2016	0.1932	0.1845	0.1668	0.1801	21	Poland	0.1718	0.1663	0.1560	0.1646	0.1607	0.1473
7	Denmark	0.5344	0.5225	0.5224	0.5245	0.5236	0.4772	22	Portugal	0.3086	0.2842	0.3564	0.5373	0.2805	0.3134
8	Estonia	0.3581	0.3845	0.3863	0.3863	0.3935	0.3923	23	Romania	0.1325	0.1149	0.1175	0.1279	0.1234	0.1103
9	Finland	0.8102	0.6320	0.6518	0.8002	0.3830	0.6527	24	Slovak	0.2098	0.2262	0.2211	0.2255	0.2178	0.2051
10	France	0.5133	0.5118	0.5155	0.5151	0.5147	0.4693	25	Slovenia	0.3133	0.3118	0.3111	0.3158	0.3124	0.3074
11	Germany	0.5810	0.5769	0.4709	0.6121	0.5965	0.5579	26	Spain	0.3755	0.3601	0.3310	0.3547	0.3343	0.3190
12	Greece	0.8982	0.5561	0.5949	0.5891	0.5941	0.6248	27	Sweden	0.8569	0.8487	1	1	0.4086	0.8137
13	Hungary	0.1782	0.1721	0.1809	0.1800	0.1710	0.1604	28	United Kingdom	0.5196	0.5191	0.5228	0.5225	0.5213	0.4671
14	Ireland	0.5346	0.5345	0.5371	0.5408	0.5442	0.4868	Average in EU		0.4774	0.4313	0.4384	0.4668	0.4198	0.4276
15	Italy	0.9077	0.5308	0.4815	0.6925	0.8548	0.6928								

^1^ Decision Making Unit.

**Table 6:** Overall efficiency by non-EU countries from 2010 to 2014.

No.	DMU	2010	2011	2012	2013	2014	Annual Average	No.	DMU	2010	2011	2012	2013	2014	Annual Average
1	Albania	0.6137	0.6052	0.6220	0.6253	0.6283	0.5342	28	Kyrgyz	0.6202	0.4412	0.3579	0.3423	0.3520	0.3456
2	Algeria	0.1942	0.2243	0.2142	0.2387	0.2922	0.2050	29	Malaysia	0.1846	0.1824	0.1679	0.1672	0.1568	0.1623
3	Argentina	0.1760	0.5447	0.2203	0.1942	0.1598	0.2194	30	Mexico	0.1981	0.1932	0.1863	0.2026	0.1909	0.1721
4	Australia	1	0.7938	0.5463	0.5482	0.5544	0.6637	31	Mongolia	1	0.4363	0.3377	0.3674	0.3815	0.4446
5	Bangladesh	0.2024	0.1844	0.1932	0.1966	0.1923	0.1977	32	Morocco	0.1550	0.1421	0.1268	0.1335	0.1366	0.1378
6	Belarus	0.1094	0.1248	0.1123	0.1200	0.1211	0.1190	33	Nepal	0.6480	0.6417	0.6342	0.6317	0.6218	0.5656
7	Botswana	0.1988	0.1870	0.2156	0.2319	0.2337	0.2144	34	New Zealand	0.7662	0.7806	0.7998	0.5886	0.8050	0.7364
8	Brazil	0.2380	0.2441	0.2223	0.2415	0.2153	0.1847	35	Nigeria	0.6752	0.6660	0.6282	0.6284	0.6274	0.4691
9	Brunei	1	1	1	1	1	1	36	Norway	1	0.5210	0.5219	0.5258	0.5260	0.5798
10	Cambodia	0.5109	0.6223	0.4937	0.4455	0.4700	0.5100	37	Pakistan	0.2217	0.2831	0.2861	0.2178	0.1868	0.2508
11	Cameroon	0.1973	0.1608	0.2049	0.2451	0.2718	0.2112	38	Peru	0.1505	0.1283	0.1731	0.1536	0.1303	0.1317
12	Canada	0.8287	0.8233	0.4579	0.8264	0.8222	0.7422	39	Philippines	0.1486	0.1554	0.1871	0.1787	0.1543	0.1505
13	Chile	0.1732	0.1526	0.1907	0.1590	0.1455	0.1485	40	Russian	0.1448	0.1742	0.1944	0.2136	0.1927	0.1457
14	China	0.1098	0.1203	0.1333	0.1478	0.1398	0.1245	41	Saudi Arabia	1	1	1	1	1	1
15	Colombia	0.1841	0.1596	0.2021	0.1941	0.1665	0.1519	42	Serbia	0.1481	0.1685	0.1585	0.1681	0.1565	0.1562
16	Costa Rica	0.2579	0.2596	0.3080	0.2787	0.2527	0.2580	43	Singapore	1	1	1	1	1	1
17	Cuba	0.5887	0.5930	0.6171	0.5786	0.5459	0.5441	44	South Africa	0.1241	0.1195	0.1225	0.1034	0.0909	0.0715
18	Georgia	0.4444	0.7162	0.4121	0.3884	0.3323	0.4525	45	Sri Lanka	0.2168	0.2073	0.2060	0.1949	0.1974	0.2038
19	Iceland	1	1	1	1	1	1	46	Switzerland	0.5177	0.5149	0.5202	1	1	0.6872
20	India	0.0690	0.0595	0.0604	0.0609	0.0555	0.0582	47	Thailand	0.0843	0.0722	0.0789	0.0818	0.0700	0.0702
21	Iran	0.1969	0.2160	0.2138	0.1819	0.0908	0.1686	48	Tunisia	0.1821	0.1756	0.1586	0.1569	0.1581	0.1655
22	Iraq	0.2903	0.7368	1.0000	1.0000	0.2420	0.5941	49	Turkey	0.2091	0.1742	0.1780	0.1988	0.1839	0.1602
23	Israel	0.8999	0.4283	0.4939	0.5991	0.5964	0.5802	50	Ukraine	0.0775	0.1029	0.1174	0.0956	0.0805	0.0895
24	Japan	1	1	1	1	1	1	51	United Arab Emirates	0.8872	1	1	1	1	0.9774
25	Kazakhstan	0.1328	0.1667	0.1883	0.2371	0.1949	0.1647	52	United States	1	1	1	1	1	1
26	Kenya	0.1442	0.1376	0.1904	0.1851	0.1720	0.1600	53	Vietnam	0.0605	0.0558	0.0631	0.0586	0.0594	0.0584
27	Korea, Rep.	1	1	1	1	0.4335	0.8662	Average in non-EU		0.4374	0.4264	0.4098	0.4214	0.3922	0.3963

**Table 7:** Average overall efficiency in EU and non-EU countries from 2010 to 2014 in each stage.

Countries	2010-I	2011-I	2012-I	2013-I	2014-I	Stage I
EU Countries (28)	0.6165	0.6159	0.6349	0.6659	0.6159	0.6298
Non-EU Countries (53)	0.4532	0.4679	0.4633	0.4673	0.4362	0.4576
Countries	2010-II	2011-II	2012-II	2013-II	2014-II	Stage II
EU Countries (28)	0.3382	0.2466	0.242	0.2676	0.2237	0.2636
Non-EU Countries (53)	0.4216	0.3848	0.3562	0.3754	0.3482	0.3772

**Table 8:** Average overall TGRs of EU and non-EU countries from 2010 to 2014.

Countries	2010	2011	2012	2013	2014	Total
EU Countries (28)	0.7234	0.3485	0.345	0.3641	0.3506	0.6592
Non-EU Countries (53)	0.8739	0.8625	0.8574	0.8554	0.8743	0.8524
Countries	2010(I)	2011(I)	2012(I)	2013(I)	2014(I)	stage(I)
EU Countries (28)	0.895	0.886	0.878	0.919	0.889	0.889
Non-EU Countries (53)	0.874	0.871	0.863	0.858	0.883	0.861
Countries	2010(II)	2011(II)	2012(II)	2013(II)	2014(II)	stage (II)
EU Countries (28)	0.478	0.386	0.387	0.408	0.382	0.406
Non-EU Countries (53)	0.875	0.848	0.860	0.849	0.855	0.853

**Table 9:** Comparison of energy efficiencies during 2010 to 2014.

Year	Countries	Non-Renewable Energy	Renewable Energy	Labor	GDP	CO_2_	PM2.5
2010	Non-EU	0.3992	0.5866	0.4318	0.9562	0.5902	0.7609
	EU	0.4336	0.7302	0.6860	0.9998	0.7464	0.8920
2011	Non-EU	0.4139	0.5760	0.4590	0.9500	0.5956	0.7504
	EU	0.4779	0.6984	0.6754	0.9979	0.6910	0.8217
2012	Non-EU	0.3866	0.5953	0.4430	0.9610	0.5856	0.7593
	EU	0.5337	0.6786	0.6931	0.9987	0.6801	0.8837
2013	Non-EU	0.4286	0.5827	0.4398	0.9519	0.5847	0.7865
	EU	0.5772	0.7138	0.7069	1.0000	0.7167	0.9165
2014	Non-EU	0.4048	0.5668	0.4296	0.9276	0.5532	0.7272
	EU	0.5149	0.6703	0.6709	0.9929	0.6629	0.9318
Annual average	Non-EU	0.4066	0.5815	0.4406	0.9493	0.5819	0.7568
	EU	0.5075	0.6982	0.6864	0.9979	0.6994	0.8891

**Table 10:** Comparison of health efficiencies during 2010 to 2014.

Year	Countries	Health Expenditure	Tuberculosis Rate	Mortality Rate of Children	Mortality Rate of the Adult	Survival Rate of 65 Years Old
2010	Non-EU	0.4688	0.5940	0.7322	0.7036	0.9307
	EU	0.3565	0.7421	0.8932	0.6784	1.0000
2011	Non-EU	0.4358	0.6353	0.7201	0.7139	0.9132
	EU	0.2703	0.7357	0.8550	0.6602	1.0000
2012	Non-EU	0.4150	0.5503	0.6861	0.6881	0.9190
	EU	0.2709	0.6246	0.8397	0.6584	1.0000
2013	Non-EU	0.4267	0.5633	0.7188	0.7045	0.9281
	EU	0.2954	0.6148	0.8101	0.6921	0.9966
2014	Non-EU	0.4025	0.5237	0.6662	0.7013	0.9176
	EU	0.2506	0.5613	0.7913	0.7276	1.0000
Annual Average	Non-EU	0.4298	0.5733	0.7047	0.7023	0.9217
	EU	0.2887	0.6557	0.8378	0.6834	0.9993

**Table 11:** Comparison of the annual health efficiencies of EU countries from 2010 to 2014.

No.	Country	Health Expenditure	Children’s Mortality Rate	Adult’s Mortality Rate	No.	Country	Health Expenditure	Children’s Mortality Rate	Adult’s Mortality Rate
1	Austria	0.0520	0.6284	0.7301	15	Hungary	0.0987	0.6981	0.3218
2	Belgium	0.0509	0.6881	0.6753	16	Ireland	0.0959	0.6532	0.7720
3	Bulgaria	0.1812	0.7185	0.8879	17	Italy	0.5189	0.9906	1
4	Cyprus	1	1	1	18	Lithuania	0.2057	1	0.2700
5	Czech	0.0875	0.8439	0.5261	19	Luxembourg	0.4514	0.9881	0.8409
6	Germany	0.2883	0.9477	0.8147	20	Latvia	0.3255	1	0.4479
7	Denmark	0.0621	0.6801	0.6829	21	Malta	1	1	1
8	Spain	0.0594	0.7769	0.8474	22	Netherlands	0.0941	0.8085	0.8911
9	Estonia	0.6073	1	0.3787	23	Poland	0.0603	0.7638	0.4151
10	Finland	0.8337	1	0.7984	24	Portugal	0.0972	0.7801	0.6238
11	France	0.034	0.7863	0.6556	25	Romania	0.0504	0.8139	0.4553
12	UK	0.0529	0.6383	0.7507	26	Slovak	0.1273	0.5693	0.4153
13	Greece	0.3667	0.8626	0.7748	27	Slovenia	0.2918	1	0.6500
14	Croatia	0.1693	0.8231	0.5096	28	Sweden	0.8224	1.0000	0.9986

**Table 12:** Comparison of the annual health efficiencies of non-EU countries from 2010 to 2014.

No.	Country	Health Expenditure	Children Mortality Rate	Adult Mortality Rate	No.	Country	Health Expenditure	Children Mortality Rate	Adult Mortality Rate
1	Albania	1.0000	1.0000	1.0000	28	Kyrgyz	0.8565	0.7293	0.5650
2	United Arab	1.0000	1.0000	1.0000	29	Cambodia	0.9639	1.0000	0.7213
3	Argentina	0.0684	0.2926	0.4262	30	Korea	0.8391	1.0000	0.9709
4	Australia	0.3975	0.7529	0.9098	31	Sri Lanka	0.2518	0.8540	0.5110
5	Bangladesh	0.3219	0.7827	0.9176	32	Morocco	0.1760	0.4164	0.9844
6	Belarus	0.1800	1.0000	0.2221	33	Mexico	0.0844	0.3374	0.5672
7	Brazil	0.1061	0.2742	0.6183	34	Mongolia	0.8930	0.8241	0.5203
8	Brunei	1.0000	1.0000	1.0000	35	Malaysia	0.1547	0.9007	0.4357
9	Botswana	0.2393	0.9567	0.3831	36	Nigeria	0.8526	0.3859	0.9613
10	Canada	0.8694	0.9276	1.0000	37	Norway	0.2449	0.8754	0.8899
11	Switzerland	0.4237	0.8166	0.9894	38	Nepal	1.0000	1.0000	1.0000
12	Chile	0.0681	0.5514	0.5727	39	New Zealand	0.9521	0.8857	0.9977
13	China	0.1442	0.4750	1.0000	40	Pakistan	0.4443	0.2788	0.5954
14	Cameroon	0.2514	0.7055	0.4601	41	Peru	0.0737	0.5170	0.5721
15	Colombia	0.0560	0.2891	0.3381	42	Philippines	0.1472	0.4086	0.3576
16	Costa Rica	0.2047	0.6001	0.6115	43	Russian	0.1287	0.6390	0.4572
17	Cuba	0.2058	0.7171	0.7007	44	Saudi Arabia	1.0000	1.0000	1.0000
18	Algeria	0.1126	0.3812	0.7374	45	Singapore	1.0000	1.0000	1.0000
19	Georgia	0.8809	0.7048	0.5366	46	Serbia	0.1573	0.9893	0.4732
20	India	0.0656	0.3932	0.4526	47	Thailand	0.0400	0.7741	0.4534
21	Iran	0.1233	0.3017	0.6779	48	Tunisia	0.2029	0.6496	0.7128
22	Iraq	0.6517	0.5755	0.7211	49	Turkey	0.0465	0.3091	0.6121
23	Iceland	1.0000	1.0000	1.0000	50	Ukraine	0.0914	0.7577	0.4065
24	Israel	0.3430	0.7915	0.9403	51	United States	1.0000	1.0000	1.0000
25	Japan	1.0000	1.0000	1.0000	52	Vietnam	0.0744	0.4173	0.6167
26	Kazakhstan	0.1392	0.4842	0.2346	53	South Africa	0.0805	0.8118	0.8231
27	Kenya	0.1682	0.8128	0.5674					
